# Selective Nutrient Transport in Bacteria: Multicomponent Transporter Systems Reign Supreme

**DOI:** 10.3389/fmolb.2021.699222

**Published:** 2021-06-29

**Authors:** James S. Davies, Michael J. Currie, Joshua D. Wright, Michael C. Newton-Vesty, Rachel A. North, Peter D. Mace, Jane R. Allison, Renwick C.J. Dobson

**Affiliations:** ^1^ Biomolecular Interaction Centre and School of Biological Sciences, University of Canterbury, Christchurch, New Zealand; ^2^ Department of Biochemistry and Biophysics, Stockholm University, Stockholm, Sweden; ^3^ Biochemistry Department, School of Biomedical Sciences, University of Otago, Dunedin, New Zealand; ^4^ Maurice Wilkins Centre for Molecular Biodiscovery and School of Biological Sciences, Digital Life Institute, University of Auckland, Auckland, New Zealand; ^5^ Department of Biochemistry and Molecular Biology, Bio21 Molecular Science and Biotechnology Institute, University of Melbourne, Parkville, VIC, Australia

**Keywords:** protein-protein interaction, membrane proteins, transport mechanism, TRAP transporter, ABC transporter

## Abstract

Multicomponent transporters are used by bacteria to transport a wide range of nutrients. These systems use a substrate-binding protein to bind the nutrient with high affinity and then deliver it to a membrane-bound transporter for uptake. Nutrient uptake pathways are linked to the colonisation potential and pathogenicity of bacteria in humans and may be candidates for antimicrobial targeting. Here we review current research into bacterial multicomponent transport systems, with an emphasis on the interaction at the membrane, as well as new perspectives on the role of lipids and higher oligomers in these complex systems.

## Introduction

All bacteria must scavenge and take up nutrients from their environment to survive. The cellular repertoire of transporter proteins is responsible for both the uptake of essential nutrients such as carbohydrates, amino acids, and metals into the cell, as well as the efflux of toxins and antimicrobial agents out of the cell (Saier, 2000). Transporter proteins therefore play key roles in bacterial colonisation, pathogenesis, and antimicrobial resistance (Putman et al., 2000; Brown et al., 2008; Siegel and Weiser, 2015). In contrast to channel proteins, which catalyse the high-flux of molecules down a concentration gradient, transporters can couple uphill substrate translocation with the movement of ions down their electrochemical gradient (secondary active transporters), or by using processes such as ATP hydrolysis (primary active transporters). These processes enable bacteria to scavenge nutrients that may be scarce (Nikaido and Saier, 1992).

There are many different families of bacterial transporter proteins, with differing folds, substrate specificities, and mechanisms of transport. In this review, we focus on transporter systems that utilise a substrate-binding protein (SBP) to deliver nutrients to the membrane component. These types of transport systems are very substrate specific, which has been shown to provide a competitive advantage to pathogenic bacteria during colonisation and infection; for example, the uptake of carbohydrates such as sialic acid and fucose (Almagro-Moreno and Boyd, 2009; Ng et al., 2013), amino acids such as l-glutamate (Colicchio et al., 2009), and metal ions such as zinc (Nielubowicz et al., 2010) and iron (Perry et al., 2015). Multicomponent transporters are thought to be particularly advantageous in environments with low nutrient availability, and during different stages of infection where bacteria can upregulate transporters to suit their environments (Sanchez-Ortiz et al., 2021).

Two important families of multicomponent active transporters are of particular interest: the widely studied ATP-binding cassette (ABC) transporters and the less understood tripartite ATP-independent periplasmic (TRAP) transporters. ABC transporters are found across all kingdoms of life, with many eukaryotic ABC transporters implicated in disease states (Gerlach et al., 1986; Gros et al., 1986; Hyde et al., 1990; Higgins, 1992; Borst and Elferink, 2002; Wolf et al., 2012). The role of ABC transporters in bacterial pathogenicity is well established (Tanaka et al., 2018), and ABC classes that lack homologs in eukaryotes have been explored as potential drug targets against Gram-positive bacteria (Counago et al., 2012). In contrast, TRAP transporters are not found in eukaryotes and are only present in bacteria and archaea (Forward et al., 1997; Kelly and Thomas, 2001; Fischer et al., 2010; Mulligan et al., 2011). Moreover, TRAP transporters are important for host colonisation and persistence by pathogenic bacteria (Severi et al., 2005; Almagro-Moreno and Boyd, 2009; Jenkins et al., 2010), thus representing an attractive therapeutic target. This link between transport by TRAPs and pathogenicity is comprehensively reviewed by Rosa et al. (2018).

ABC and TRAP transporters differ considerably in sequence, structure and mechanism of transport. ABC transporters are primary active transporters that use energy from ATP binding or hydrolysis to drive large structural rearrangements of the membrane domains—in particular, rearrangement between outward- and inward-facing orientations (Locher, 2016). The mechanism of TRAP transport is unresolved, but it is clear that they operate *via* a secondary active transport mechanism, coupling target molecule transport to the movement of cations down an electrochemical gradient (Mulligan et al., 2009). Common to both of these systems is the use of a high-affinity SBP to unload substrates to the integral membrane domain (Berntsson et al., 2010; Fischer et al., 2015). In ABC transporters, the general mechanism of substrate unloading involves distortion of the SBP upon docking at the membrane domain (Locher, 2016). This rearrangement lowers the affinity of the interaction between ligand and SBP, allowing substrate release for transport. It is not yet known if this is the same for TRAP transporters.

Given their link to the pathogenesis and survival strategy of bacteria, both ABC and TRAP transporters present as interesting targets for antimicrobial development. Inhibiting the SBP is an obvious strategy that has been shown to impede bacterial growth and pathogenesis *in vivo* (Ilari et al., 2016). Inhibiting substrate binding at the membrane components, or the protein-protein interaction at the membrane are strategies to be considered. Others have considered using the SBP in a “Trojan horse” mechanism to deliver bactericidal agents into the cell (Wilson et al., 2016). Understanding the structure-function relationships of these transporters is therefore key to chemical targeting and antimicrobial design (Scalise et al., 2020). Here we review the interplay of prokaryotic ABC and TRAP transporters with their cognate SBPs, as well as new perspectives on the structure and function of these systems.

### Multicomponent Transporter Prevalence

The prevalence of multicomponent transporter genes in bacteria varies considerably. The micro-environment that the cell occupies is linked to the number and the type of transporter a bacterium may possess, with the number of transporters in the genome being generally proportional to genome size (Davidson et al., 2008). ABC transporter proteins are by far the most abundant of transporters, typically accounting for half of the transporters in a bacterial genome. *Escherichia coli* has a 4.6 Mb genome and encodes 78 ABC transporter systems, typical for a genome of this size. In contrast, *Mycobacterium tuberculosis* has a 4.4 Mb genome and encodes only 38 ABC systems, while *Agrobacterium tumefaciens* has a 5.7 Mb genome that encodes over 200 ABC systems (Davidson et al., 2008). *M. tuberculosis* lives a parasitic intracellular nutrient-rich lifestyle where the requirement to select nutrients that are low in abundance has been lost, whereas *A. tumefaciens* lives in the soil, a highly competitive environment. Recent comparative genomics of clinically significant pathogenic bacteria, such as *Salmonella enterica*, *E. coli* and species of *Bacteroides* show that ABC transporters are among the most commonly encoded transporter system, comprising 20–30% of all transporter proteins in the strains examined (Do et al., 2017; Zafar and Saier, 2018).

TRAP transporter prevalence in bacterial genomes is variable. The TransportDB 2.0 database (http://www.membranetransport.org/transportDB2/index.html) shows that out of the 2,722 prokaryotic genomes analysed in the database, 1,252 (46%) have at least one TRAP system in the genome. Some species have only one TRAP system (*E. coli 042*), others have over 20 (*Silicibacter pomeroyi DSS-3* and *Chromohalobacter salexigens DSM 3043*) (Mulligan et al., 2007; Elbourne et al., 2017). Moreover, TRAP proteins appear more common in bacteria that live in saline environments (Mulligan et al., 2007). It seems likely that these species have adapted to exploit the high sodium concentration in their surrounding environments by utilising a sodium gradient to power transport as opposed to ATP. In addition, Bergauer et al. (2018) observed that TRAP transporters are more prevalent in bacteria that live at depths greater than 500 m compared to those between 0 and 500 m. The authors hypothesise that in deep sea oligotrophic conditions, TRAP transporters are more advantageous than ABC transporters as their transport is less energy-consuming due to a reduced requirement for ATP hydrolysis.

### Multicomponent Transporter Architecture

Buried in the lipid membrane, transporter proteins are intrinsically hydrophobic and traditionally, they are difficult to isolate and characterise. Nonetheless, there have been significant improvements in membrane protein purification methodologies, particularly with the development of new detergents and membrane mimetics. Importantly, these improvements have led to an expansion in the number of membrane protein structures, although they are still under-represented in the PDB and notably, no TRAP transporter membrane protein structure has been experimentally determined.

**FIGURE 1:**
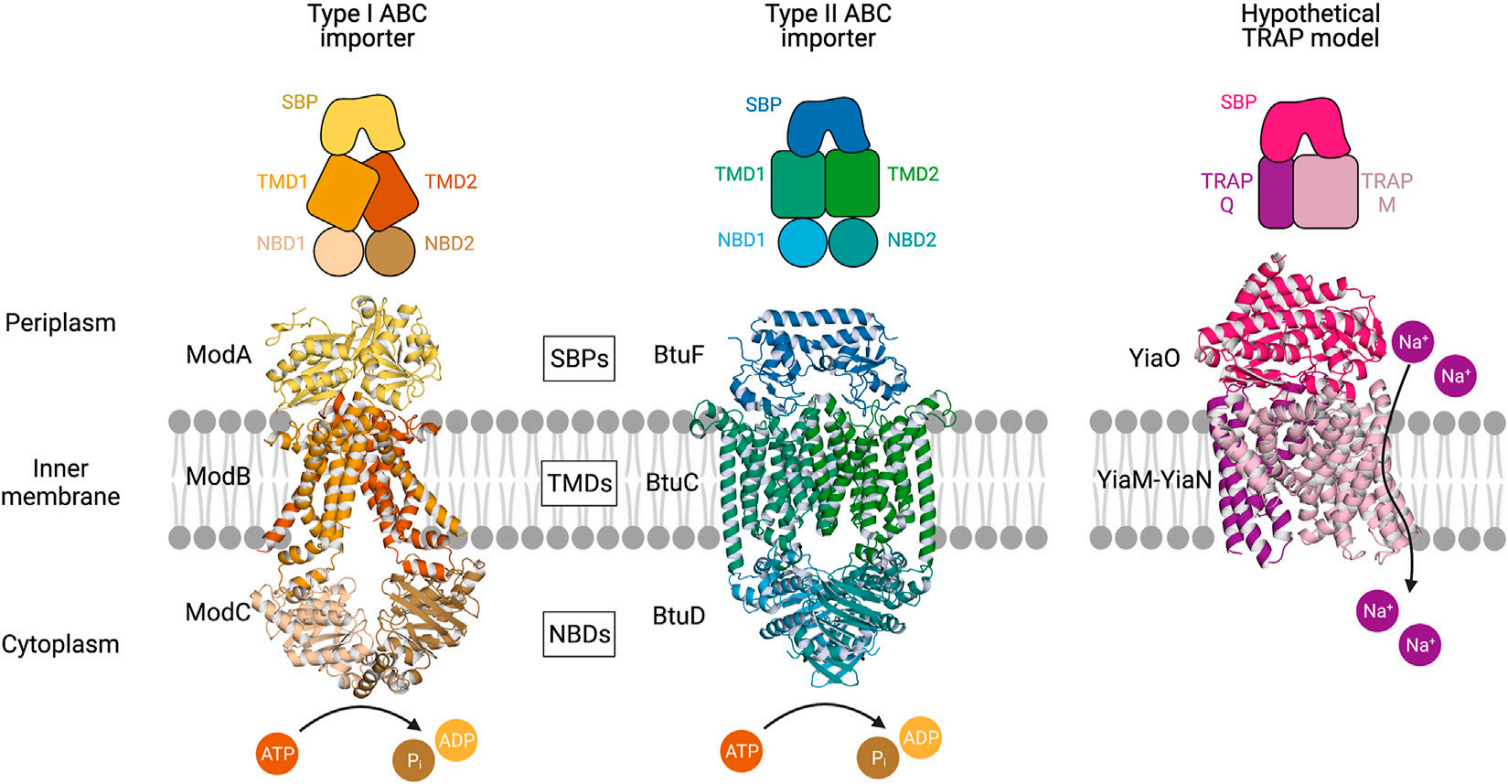
Multicomponent transporters. Left, an experimentally determined structure of the type I ABC importer ModBC-A from *A. fulgidus* (PDB ID: 2ONK). Middle, an experimentally determined structure of the type II ABC importer BtuCD-F from *E. coli* (PDB ID: 2QI9) (Hollenstein et al., 2007; Hvorup et al., 2007). Right, a hypothetical model of the TRAP transporter YiaMN-O from *E. coli* that was generated by Ovchinnikov et al. (2014) using comparative modelling in *RosettaCM*. The substrate-binding proteins (SBPs) dock to the transmembrane domains (TMDs) at the periplasmic surface of the inner membrane. ABC transporter nucleotide-binding domains (NBDs) situated in the cytoplasm catalyse the hydrolysis of ATP. The TRAP transporter facilitates the movement of sodium ions across the membrane.

*ABC transporter structure:* As of writing, 85 experimentally determined structures of ABC transporters have been deposited in the PDB, revealing wide structural diversity. Common to all ABC transporters is a dimeric membrane-bound component (homo- or hetero-) that brings together two transmembrane domains (TMDs) and two nucleotide-binding domains (NBDs), otherwise known as ATP-binding cassettes (Figure 1). The NBDs, which in some cases may be fused to a TMD, hydrolyse ATP and drive conformational changes in the transmembrane domains that in turn allow for the substrate to pass through the membrane. Recent studies suggest that either the binding, or the hydrolysis of ATP provides the “power stroke” for transport, and this varies between systems (Mishra et al., 2014; Stefan et al., 2020). NBDs are highly conserved in structure and sequence, but the TMDs are less so, which reflects the diversity in transported substrates. Historically, ABC transporters have been classified by sequence alignments and substrate specificity, which has separated the transporters into three classes. However, a surge in the number of ABC transporter structures deposited in the PDB has led others to rethink this classification system—a seven class system where ABC transporters are classified based on their transmembrane domain fold is now suggested (Thomas et al., 2020).

The TMD monomer consists of anywhere between four and ten transmembrane helices (TMH), plus a small number of connecting and coupling *α*-helices. The number and topology of helices dictate the seven ABC transporter classes that are now proposed. Classically, ABC transporters that import using an SBP are categorised as either Type I (small), with five to eight TMH per subunit, or Type II (large), with ten TMH per subunit. A coupling helix on the cytoplasmic surface interacts tightly with the NBD. Each NBD consists of two sub-domains: a larger RecA-like domain and a smaller α-helical domain that is unique to ABC transporters. Several highly conserved motifs help identify NBDs, including the Walker A and B motifs, and the signature ABC motif (Thomas and Tampé, 2020).

For Type I and Type II ABCs the periplasmic surface of the TMD serves as a docking interface for the SBP. All SBPs, including those from both ABC and TRAP systems, share a common architecture—two *α*/*β* domains linked by a hinge region about which the SBP can open and close around a ligand, which is likened to a “Venus flytrap” mechanism (Scheepers et al., 2016). Some experimental evidence favours induced-fit over conformational selection as the mechanism of substrate binding to the SBP (Gouridis et al., 2015), although it is unclear whether this is the case for all systems. This mechanism of conformational change and the conformational equilibria could both have implications for how the protein-protein interaction occurs, and the transport cycle overall.

*TRAP transporter structure:* TRAP transporters are a major class of secondary transporters used by both bacteria and archaea to import a range of carboxyl and sulfonate-containing molecules, including C4-dicarboxylates, *α*-keto acids, aromatic substrates, and amino acids (Rosa et al., 2018). TRAPs couple transport of these molecules to the movement of cations (Na^+^) down an electrochemical gradient, and also utilise a high-affinity SBP. SBPs were previously thought to be unique to ABC transporters, until the discovery of the first TRAP transporter system, dctPQM, from *Rhodobacter capsulatus* (Forward et al., 1997)*.* To date, there are no experimentally determined structures of the membrane domains of TRAP transporters, limiting our understanding. Unlike ABC systems, the TRAP membrane component is almost always heterodimeric. Typically, the TRAP membrane domains are comprised of a large or “M” domain, estimated to be 12–14 TMH with a predicted N_Out_C_Out_ topology, and a small or “Q” domain made up of 4 TMH, with an experimentally determined N_In_C_In_ topology (Wyborn et al., 2001). Together with the SBP (or P domain), they make up the “tripartite” system (Figure 1).

A number of TRAP SBPs have been characterised structurally and biochemically (Muller et al., 2006; Johnston et al., 2008; Gangi Setty et al., 2014), generally showing the canonical SBP tertiary structure. Using a structural genomics approach, Vetting et al. (2015) solved 60 high-resolution crystal structures of SBPs (46 unique) greatly expanding the knowledge base for these transporter systems.

In a small proportion of TRAP transporters, the Q and the M domains are fused together and expressed as a single polypeptide. Rarer still are fusions of the Q domain to the P domain. For the majority of TRAP transporters, these domains are transcribed separately, and oligomerise *via* an unknown mechanism to form a functional transporter (Kelly and Thomas, 2001). The large membrane domain is predicted to form the translocation channel, and is a part of the ion transporter superfamily (Rabus et al., 1999). The function of the small membrane domain is unknown, but it is hypothesised that the small domain could function as a chaperone for the folding of the large domain, or act as a landing pad for the SBP (Mulligan et al., 2011).

The best-studied members of the TRAP transporter family are the non-fused *Vibrio cholerae* and the fused (Q and M domains) *Haemophilus influenzae* SiaPQM systems. These TRAPs transport sialic acids, a family of nine-carbon amino sugars, the most common of which is *N-*acetylneuraminic acid (North et al., 2018). The focus on these systems is due to the growing interest in the role of sialic acid as an important nutrient source for pathogenic bacteria *in vivo* (North et al., 2016; North et al., 2018; Wahlgren et al., 2018; Davies et al., 2019; Coombes et al., 2020; Horne et al., 2021). Electrochemical studies have been used to characterise the transport of sialic acid through the *H. influenzae* SiaPQM system (Mulligan et al., 2009). Proteoliposome assays identified that transport by SiaQM (membrane domains) is predominantly unidirectional. Efflux of sialic acid from the proteoliposome could only be achieved with an excess of unliganded SiaP (at conditions considered unlikely to be physiologically relevant). This study also identified that at least two Na^+^ ions are coupled to the transport of sialic acid and that transport could not be driven by a pH gradient or membrane potential alone. These data strengthen the aforementioned argument that TRAP transporters have lower energetic costs in marine environments. More data is required to ascertain whether all TRAP transporters are Na^+^-dependent and have this coupling stoichiometry.

### Current Models for Protein:Protein Interactions at the Membrane

Recently, it has been shown that SBPs can adopt a wide range of conformations that can activate transport and that both transported and non-transported ligands can adopt similar conformations in solution. While the SBP is the primary specificity determinant for the target molecule, it is suggested that the fate of the transported ligand can also be determined by selectivity at the membrane domain, or by a slow opening of the SBP, preventing translocation (de Boer et al., 2019). In both situations, it is clear that the docking and allosteric interaction of these domains is key in the transport cycle. Whereas there is ample structural data to define this interaction in ABC systems, there is currently no experimentally determined structural data of the membrane components to guide our understanding of this interaction in the TRAP transporters (Figure 1).

For both ABC and TRAP transporters, docking of the SBP must trigger conformational changes in the membrane domains, and in the case of ABC transporters, catalytic transformations at the NBDs. How the soluble SBP and membrane-bound transporter interact is therefore key to understanding the transport cycle. Within the ABC transporter family, there is wide variation in how the SBP interacts with the transporter domain. The interactions have been well-characterised in the maltose-specific ABC MalFGK_2_, where it was shown that the SBP MalE interacts with the periplasmic loops of MalFG. The crystal structure of ModB_2_C_2_A (PDB ID: 2ONK) shows both SBP lobes interacting with the TMDs (Figure 1), with six salt bridges per domain (Hollenstein et al., 2007). In comparison, the BtuCD-F structure (PDB ID: 2QI9) has a very different interacting surface that reflects the asymmetric structure of the SBP, and only one salt bridge per domain (Hvorup et al., 2007).

Vigonsky et al. (2013) examined this interaction with two different Type II ABC transporters with the same substrate specificity (molybdate and tungstate), and crucially found that the interaction at the membrane is completely different between the two systems. In one system, ModBC-A from *Archaeglobus fulgidus*, the SBP appears to form a low-affinity, transient complex with the membrane domain that is stabilised by ligand binding. Contrastingly, the *H. influenzae* molybdate/tungstate ABC transporter has a high-affinity interaction that is destabilised by both ligand and nucleotide binding (Vigonsky et al., 2013). Mulligan et al. (2009) tested whether the *V. cholerae* TRAP SiaP could deliver substrate to the *H. influenzae* SiaQM membrane domains, but found no transport in a proteoliposome assay. These data together highlight the specificity of the interactions between the SBP and the membrane domains in these multicomponent systems.

Once the complex has formed, the substrate must pass to the membrane domain. The general model for this involves the disruption or distortion of the SBP high-affinity binding pocket by loops of the membrane domain. In ABC transporters, there is variation in the affinity for substrates within the membrane domain. Local concentrations of substrate are important to consider here, while the temporary binding pocket has only moderate affinity, with a *K*_*d*_ in the mM range—substrate concentration in the tunnel is thought to be at least two orders of magnitude greater than this. In Type II transporters, such as the well-studied *E. coli* vitamin B12 transporter BtuCD-F (PDB ID: 2QI9), there is no substrate binding pocket, and the substrate is released into a hydrophobic pocket with no measurable affinity—likened to a “Teflon” cavity. The interactions within the BtuCD-F system, as well as the Type I ModBC-A transporter, have been studied using surface plasmon resonance and single-molecule fluorescence resonance energy transfer (FRET), and more recently by native mass spectrometry (Fiorentino et al., 2019). This variation in interaction perhaps reflects the diversity of both SBP folds and, as elaborated below, general mechanisms of transport.

### Mechanisms of Transport

The alternating-access model is the dominant descriptor of substrate transport for ABC transporters (Locher, 2016). In its simplest form, this model involves conformational changes in the membrane domains that expose the substrate-binding site to either side of the membrane, which is achieved through an allosteric coupling of intracellular and extracellular gates within the transporter. The alternating-access model can be further divided into three distinct types: the rocker-switch, the rocking-bundle (or gated-pore) and the elevator model (Drew and Boudker, 2016). For descriptive purposes and in brief, if a typical transporter protein is described as two bundles (with an N- and a C-terminal domain), structurally similar bundles rearrange in a symmetrical fashion around a central substrate-binding site in the rocker-switch model; structurally dissimilar bundles rearrange asymmetrically around a central substrate-binding site in the rocking-bundle model; and in the elevator mechanism the two bundles are highly divergent, with one of them remaining fixed and immobile within the membrane, while the other moves against this bundle to physically translocate the substrate to the other side of the membrane in an elevator-type fashion (Drew et al., 2021).

A model of transport by TRAP transporters has been proposed and is based upon the alternating-mechanism seen in Type I ABC transporters (Mulligan et al., 2009). Recent work has started to uncover potential “scoop loops” in the membrane domains of TRAP transporters (Peter et al., 2021). Additionally, Darby et al. (2019) have found that disruption of the ordered waters within the binding cleft of an SBP can dramatically alter substrate binding affinity. They discovered that a mutation made on the surface of the protein was able to severely disrupt ligand binding at the distal (∼4.7 Å) binding site. This may hint at how the membrane domains allosterically modulate the SBP—a subtle interaction at the surface of the binding protein may be all that is required to trigger the release of the substrate. This type of interaction fits with experiments performed by Marinelli et al. (2011), where the authors constructed an SBP mutant that is biased toward an open conformation, which in turn had a markedly lower affinity for substrate—providing some evidence that the binding affinity may be allosterically modulated. Recently, crystal structures of the *V. cholerae* SiaP, combined with single-molecule FRET experiments have shown that the conformational change is primarily substrate-induced (Glaenzer et al., 2017). More experimental evidence is required to better understand the transport cycle, particularly how the loop regions of the TRAP transporter interact with the SBP, and in general, how TRAP transporters function—this is a key knowledge gap in the field.

### Co-Evolution and its Potential for Defining Substrate-Binding Protein:Membrane Protein Interactions

An emerging tool for understanding multicomponent transporter systems is the analysis of co-varying residues. In particular, new statistical methods now allow for an accurate prediction of co-varying residues, in turn thought to be co-evolving. These residue pairs are a very good predictor of spatial proximity (Marks et al., 2011; Jones et al., 2012; Kamisetty et al., 2013). This is of particular interest in the case of both ABC and TRAP transporters, where the subunits are functionally connected and appear to operate independently of other proteins (Juan et al., 2008). The abundance of sequences available for both families make them suitable for this kind of analysis.

**FIGURE 2:**
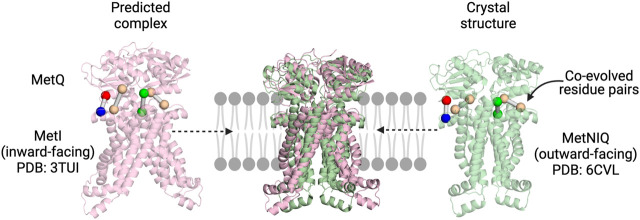
Co-evolution analysis as a tool to explore SBP:TMD interactions in multicomponent systems. The predicted MetIQ structure (Ovchinnikov et al., 2014) maps well on to the MetNIQ crystal structure (MetN not displayed) (Nguyen et al., 2018). The top co-evolved residues between the two components are in similar positions, with the predicted complex correctly orienting the SBP.

Co-evolution has been successfully used to predict the SBP:TMD complex of an ABC transporter. Ovchinnikov et al. (2014) docked the SBP (MetQ) to the TMDs (MetI) of the *E. coli* methionine transporter MetNIQ using co-evolution restraints generated by the *GREMLIN* tool. At the time, the structure of the MetNIQ complex had not been determined, but has since been solved (PDB ID: 6CVL). Strikingly, a comparison of these two structures shows the remarkable accuracy of these predictions (Figure 2). The accuracy of this method was confirmed using a benchmark set, where nearly all identified co-varying residues were in contact in the already solved complex structure. Co-evolution analysis was used to both inform the building of a comparative model of the TRAP transporter Q and M domains using *Rosetta*, as well as the docking of the P domain (Figure 1) (Ovchinnikov et al., 2014). This model, although yet to be verified experimentally, gives us the first structural picture of the membrane domains, and may be of use to inform mechanistic experiments.

### Importance of Lipids for Multicomponent Transporters

It is apparent that the lipid environment within which a membrane protein transporter is embedded plays an important role in modulating stability and activity. There are a wide variety of membrane mimetics currently available for transporter purification and characterisation, with different levels of similarity to the cell membrane. These include micelles, bicelles, peptidiscs, saposins, amphipols, styrene maleic acid lipid particles (SMALPs), nanodiscs, and liposomes (Chorev and Robinson, 2020). Each mimetic can result in substantially different transporter conformations and activities compared to the native environment of the protein. There are several examples of ABC transporters displaying significantly higher activity and/or stability when reconstituted into lipid environments, such as nanodiscs or liposomes, compared to their apparent activity when measured in detergent micelles. Examples of this include Wzm-Wzt, P-gp, and MalFGK_2_ (O’Mara and Mark, 2012; Bao et al., 2013; Bi et al., 2018). O’Mara and Mark (2012) carried out molecular dynamics simulations using the crystal structure of P-gp and found that this conformation of P-gp was stable, yet when inserted into model membranes the structure quickly deformed.

The bacterial ABC transporter MsbA, which can function both as a lipid flippase and a drug transporter, adopts a different conformation in lipid nanodiscs (*via* cryo-EM) than that observed when crystallised with detergent (Ward et al., 2007; Mi et al., 2017). Studies on MsbA comparing structure and activity in detergent to that in nanodiscs or liposomes observed the lowest activity in detergent (Kawai et al., 2011; Arana et al., 2019). MsbA is also significantly more stable in lipid carrier systems when compared MsbA in detergent or amphipol (Kehlenbeck et al., 2019). Although there is no substitute for native lipids, some detergents appear to be better at retaining functional activity, such as the maltose-neopentyl glycol (MNG) class. These are mild detergents that better mimic the classic two-tailed lipid structure, with a quaternary “linking” carbon that restricts the conformational flexibility of the molecule (Chae et al., 2010). Compared to another commonly used detergent, n-Dodecyl *β*-D-maltoside (DDM), MNG is predicted to pack more efficiently around the transmembrane region (Lee et al., 2020).

The composition of the lipid membrane varies between bacteria and can also change with their environment. Common lipids include phosphatidylglycerol (PG), cardiolipin (CL), phosphatidylethanolamine (PE) as well as methylated derivatives of PE such as phosphatidylcholine (PC) (Sohlenkamp and Geiger, 2016). Furthermore, the distribution of lipids within the bilayer is asymmetric. There are several studies comparing transporter activity in PC and PE lipids (Ahn et al., 2000; Gustot et al., 2010; Bao et al., 2013; Rice et al., 2014). One key observation here is that compared to the quaternary ammonium head groups of PC, the primary ammonium head groups of PE have greater hydrogen bonding potential and form specific interactions with transporter amino acid side chains (Immadisetty et al., 2019).

With the known importance of lipids for transporter function, it is essential that new methods are developed for their identification and for determining their role in transporter function. Native mass spectrometry is emerging as a valuable technique to investigate protein-lipid interactions. Typical identification of required lipids and their interactions with transporters centre around *in vivo* studies, X-ray crystallography, and cryo-EM, which are challenging, time-consuming, and often low resolution, not allowing for proper tail and head identification (Bolla et al., 2019). High-energy native mass spectrometry has been gaining momentum in the field to identify lipids linked to function. Using a mild detergent for the purification of the ABC transporter TmrAB, successive delipidation, mass spectrometry, and ATPase assays showed a subset of closely associated lipids remain after detergent solubilisation. Critically, as more lipids were extracted from TmrAB, evidenced from the mass spectra, the corresponding ATPase activity decreased (Bechara et al., 2015).

The role of lipids in the assembly and function of the TRAP family is yet to be established but it is conceivable that interfacial lipids may play some role in the stability of the heterodimeric complex.

### Oligomeric State and Implications for Function

Transporters, especially those that use an SBP, are typically functional as single transporter units. The possibility of larger assemblies occurring in the crowded environment of the membrane should be considered, where it is estimated that the protein composition may be as high as 30–55% of the membrane area (Linden et al., 2012). These assemblies may function independently of each other, or the conformational changes may be linked between protomers. Many studies do not take place in the membrane, or with measurement of oligomeric state in mind. There have been cases of higher-order ABC transporter oligomerisation (tetramers and above), but as most structural work is performed in detergents, physiologically relevant oligomerisation may be missed. Several biophysical techniques can accurately determine transporter oligomeric state in detergent, but in these experiments, it can be difficult to discriminate between cohabitation in the micelle and biologically relevant oligomerisation. Recently, the dicarboxylate transporter VcINDY from *V. cholerae* (which is of the divalent anion-sodium symporter family) has been shown to form dimers, although transport *via* an elevator-type mechanism does not appear to be coupled between protomers (Mancusso et al., 2012; Mulligan et al., 2016). The biological relevance of this behaviour is not yet clear.

In ABC transporters, dimerisation of two subunits generates the active transporter core that binds the cognate SBP. Higher-order oligomers of the transporter core have been implicated in the function of several families of mammalian ABC transporters, but less is known about the oligomeric status in bacteria. One example is the *M. tuberculosis* ABC transporter Rv1747, which is important for *M. tuberculosis* growth in hosts and has recently been found to form higher-order assemblies termed “nanoclusters” at the membrane (Heinkel et al., 2019). Super-resolution microscopy was used to observe these clusters, where it appears the clustering is driven by oligomerisation and phase separation behaviour of a cytoplasmic regulatory module. The role of transporter oligomerisation in this clustering is unclear, although ABC transporter oligomerisation and subsequent co-localisation has been reported for human systems. Oligomerisation may conceivably improve transport efficiency *via* cooperativity, which has been seen in ABC transporters where an SBP is fused to the transport domain (Biemans-Oldehinkel and Poolman, 2003).

Recently, several ABC SBPs from the bacterium *Thermotoga maritima* have been identified to form homodimers that act as an allosteric switch (Li et al., 2017). These dimers dissociate into monomers upon ligand binding as a proposed form of transporter regulation. Another SBP from *T. maritima*, the arginine binding protein TmArgBP, is anchored to the membrane and forms a C-terminal helix-swapped dimer that could simultaneously interact with two ABC transporter cores (Ruggiero et al., 2014).

It is not known whether oligomerisation can occur with the integral membrane component of TRAP transporters (e.g., heterotetramers, where the heterodimer species oligomerise) as the structure is undetermined, although dimeric TRAP SBPs have been structurally characterised by crystallography (Gonin et al., 2007; Vetting et al., 2015). These dimeric SBPs (of which many homologs are predicted by sequence) have an extended C-terminal helix away from the ligand-binding site that swaps over to form a dimer, positioning the binding sites in a back-to-back arrangement. Although it may be predicted, no cooperative binding was seen in the first example of these dimeric SBPs, TakP (Gonin et al., 2007). Any functional advantages of dimeric SBPs are still unknown, although dimerisation could conceivably increase transport efficiency, or be a part of the mechanism.

Our current examples of multicomponent transporters are functional without forming larger assemblies, but there are examples of SBP oligomerisation. This area has not expanded much over the last decade, potentially due to the difficulty of studying these systems. Examples could appear with future work in lipid systems such as nanodiscs.

## Conclusion

Both the TRAP and ABC transporter systems enable bacteria to selectively import nutrients and are therefore important for colonisation and persistence. While much is known about ABC transporters, from how they bind and interact with substrate-binding proteins, to the conformational transitions of the membrane and nucleotide-binding domains, relatively little is known about TRAP transporters. It is clear that in ABC transporter systems there are a number of different mechanisms of transport, and it is not a case of one-size-fits-all. The current model of the TRAP transport cycle needs further experimental testing, with the key knowledge gap being that there are no experimentally determined structures of the membrane domains. Further research into how these transporter systems function in the membrane environment is also required.
